# SNF472, a novel inhibitor of vascular calcification, could be administered during hemodialysis to attain potentially therapeutic phytate levels

**DOI:** 10.1007/s40620-018-0471-9

**Published:** 2018-01-19

**Authors:** Joan Perelló, M. Gómez, M. D. Ferrer, N. Y. Rodríguez, C. Salcedo, J. M. Buades, M. M. Pérez, J. V. Torregrosa, E. Martín, F. Maduell

**Affiliations:** 1 Research and Development Department, Laboratoris Sanifit SL., 07121 Palma de Mallorca, Spain; 2 Laboratory of Renal Lithiasis Research, IUNICS, University of the Balearic Islands, 07122 Palma, Spain; 3 Nephrology and Renal Transplantation, Hospital Clínic, Barcelona, Spain; 4 Departament de Biologia Fonamental i Ciències de la Salut, University of the Balearic Islands, 07122 Palma, Spain; 5 Nephrology Service, Hospital Son Llàtzer, Palma de Mallorca, Spain; 6 Kinrel, Madrid, Spain

**Keywords:** Cardiovascular calcification, End-stage renal disease, Hemodialysis, Phytate

## Abstract

**Background:**

Cardiovascular calcification (CVC) is a major concern in hemodialysis (HD) and the loss of endogenous modulators of calcification seems involved in the process. Phytate is an endogenous crystallization inhibitor and its low molecular mass and high water solubility make it potentially dialyzable. SNF472 (the hexasodium salt of phytate) is being developed for the treatment of calciphylaxis and CVC in HD patients. We aimed to verify if phytate is lost during dialysis, and evaluate SNF472’s behaviour during dialysis.

**Methods:**

Dialyzability was assessed *in vitro* using online-hemodiafiltration and high-flux HD systems in blood and saline. SNF472 was infused for 20 min and quantified at different time points.

**Results:**

Phytate completely dialyzed in 1 h at low concentrations (10 mg/l) but not when added at 30 or 66.67 mg/l SNF472. In bypass conditions, calcium was slightly chelated during SNF472 infusion but when the system was switched to dialysis mode the calcium in the bath compensated this chelation.

**Conclusion:**

Phytate dialyses with a low clearance. The administration of SNF472 as an exogenous source of phytate allows to attain supra-physiological levels required for its potential therapeutic properties. As SNF472 is infused during the whole dialysis session, the low clearance would not affect the drug’s systemic exposure.

## Introduction

Chronic kidney disease (CKD) is a serious condition associated with significant morbidity, premature mortality and decreased quality of life [1]. An improvement in renal therapy has been achieved in recent years by the addition of convective processes to the dialyzer, such as the on-line hemodiafiltration (OL-HDF) treatments [2, 3]. The overpressure applied to the dialyzer enhances the removal of plasmatic water, promoting the depuration of toxins by solvent drag. The excess of extracted intravascular water is replaced by means of a substitution flow (Qi ml/min), obtained from the dialysate itself. OL-HDF has demonstrated that it can deliver the most efficient removal treatment over a wide range of molecular weight (MW) uremic toxins [4] as well as improve survival [5] and quality of life.

Advanced and end-stage renal disease (ESRD) are conditions strongly associated with progressive cardiovascular calcification (CVC) [6–9]. CVC is triggered by disturbances in Ca and P metabolism and by the down-regulation and loss of endogenous inhibitors of calcification such as pyrophosphate [10], phytate [11], fetuin-A [12] and matrix-Gla protein [13]. A decrease in the concentrations of these endogenous inhibitors may contribute to functional impairments in CKD [14, 15]. In fact, the development of CVC is especially enhanced in patients undergoing hemodialysis (HD), in which crystallization inhibitors seem to be lost through the dialysis system [10].

Phytate (myo-inositol hexaphosphate) is a naturally-occurring substance found in cereals and other high-fiber foods, but it is also present in mammalian cells and tissues at concentrations in the µM range [16] and a link between phytate and health has been established, particularly with respect to calcium related diseases, such as renal stones [17–19], osteoporosis [20–22] and CVC [11, 23–25]. Phytate has been detected in human plasma with mean endogenous levels around 0.5 mg/l [26]. The low MW of phytate and its high water solubility make it potentially dialyzable and the loss of this inhibitor of crystallization during HD could additionally exacerbate the development of CVC in HD patients, as happens with pyrophosphate and bisphosphonates [10, 27]. SNF472 (an intravenous formulation of hexasodium phytate) is in active development as a novel experimental drug for the treatment of calciphylaxis and the attenuation of CVC progression in HD patients. In contrast to natural phytate, which is found in the form of its calcium-magnesium salt (also known as phytin), SNF472 consists of the hexasodium salt of phytate. The advantages of this alternative salt formulation are first its solubility (hexasodium phytate solubility in aqueous solution is > 300 g/l, while calcium-magnesium phytate is highly insoluble) and, second, that the compound can be administered without additional alterations of the calcium imbalance in HD patients. Its intended posology is intravenous infusion during dialysis, in order to obtain supra-physiological phytate plasma concentrations which would exert its therapeutic activity inhibiting CVC. Therefore, the goals of this study were to evaluate if phytate is lost through dialysis and to evaluate SNF472 behaviour through the whole dialysis circuit during the process of HD and OL-HDF.

## Materials and methods

### Experimental design

Whole blood was obtained from volunteer patients from the Haematology departments of the Hospital Clínic and Hospital Son Llàtzer (Mallorca, Spain) that were periodically undergoing therapeutic bleeding. A total of 1 L of whole blood was used in every experiment, so the blood from at least three compatible volunteers was collected per experiment forming a pool. The study was conducted in accordance with the guidelines written in the Declaration of Helsinki and all procedures involving human subjects were approved by the Ethical Committee of Clinical Investigation of the Balearic Islands. Written informed consent was obtained from all subjects.

A total of 14 different experiments were performed, 10 of them in blood and 4 in saline (Table 1). The dialysability of SNF472 was tested using three infused concentration levels (10, 30 and 66.67 mg/l) in HD and OL-HDF systems. The possible interactions of SNF472 with the dialysis system were assessed in bypass conditions, in which the blood moves through the system without filtration. The experiments were performed using the FX60-Cordiax PS membrane. FX60-Cordiax is a high-flux dialyzer with the following characteristics: helixone membrane, surface 1.4 m^2^, ultrafiltration coefficient 47 ml/h/mmHg, K_0_A Urea 1164 ml/min, steam sterilization, B2-microglobulin sieving coefficient 0.9, myoglobin sieving coefficient 0.5, and albumin sieving coefficient < 0.001. Basal clearances of cytochrome c, creatinine and urea (QB 300 ml/min) are 96, 252 and 271, respectively. One experiment was performed using a more adsorptive BG-2.1U PMMA membrane. A longer session of a total of 4 h with 30 mg/l SNF472 was also performed in saline in order to confirm the low clearance observed at low SNF472 doses. The possible effect of protein binding was assessed by comparing SNF472 dialysability in blood and in saline, while the possible effect of SNF472-calcium aggregates formation was assessed by comparing dialysability in the presence and absence of calcium in the dialysis bath. In these latter conditions, the absence of calcium was compensated by a mixture of glucose, acetic acid, sodium chloride and saline in order to mimic the osmolality and conductivity of the conventional used concentrate and avoid haemolysis.

**Table 1:** Characteristics of the experiments performed

ID	System	Concentration	Matrix	Calcium	Membrane
1	OL-HDF	66.6 mg/l	Blood	Yes	PS
2	HD	66.6 mg/l	Blood	Yes	PS
3	OL-HDF	66.6 mg/l	Blood	Yes	PMMA
4	HD	66.6 mg/l	Blood	No	PS
5	Bypass	66.6 mg/l	Blood	Yes	PS
6	OL-HDF	30 mg/l	Blood	Yes	PS
7	HD	30 mg/l	Blood	Yes	PS
8	OL-HDF	10 mg/l	Blood	Yes	PS
9	HD	10 mg/l	Blood	Yes	PS
10	Bypass	10 mg/l	Blood	Yes	PS
11	Bypass	30 mg/l	Saline	Yes	PS
12	OL-HDF	30 mg/l	Saline	Yes	PS
13	OL-HDF	30 mg/l (bolus)	Saline (5 L; 4 h)	Yes	PS
14	HD	30 mg/l	Saline	No	PS

HD: hemodialysis; OL-HDF: on-line hemodiafiltration; PMMA: polymethylmethacrylate; PS: polysulfone

### In vitro dialysis sessions

**Fig. 1:**
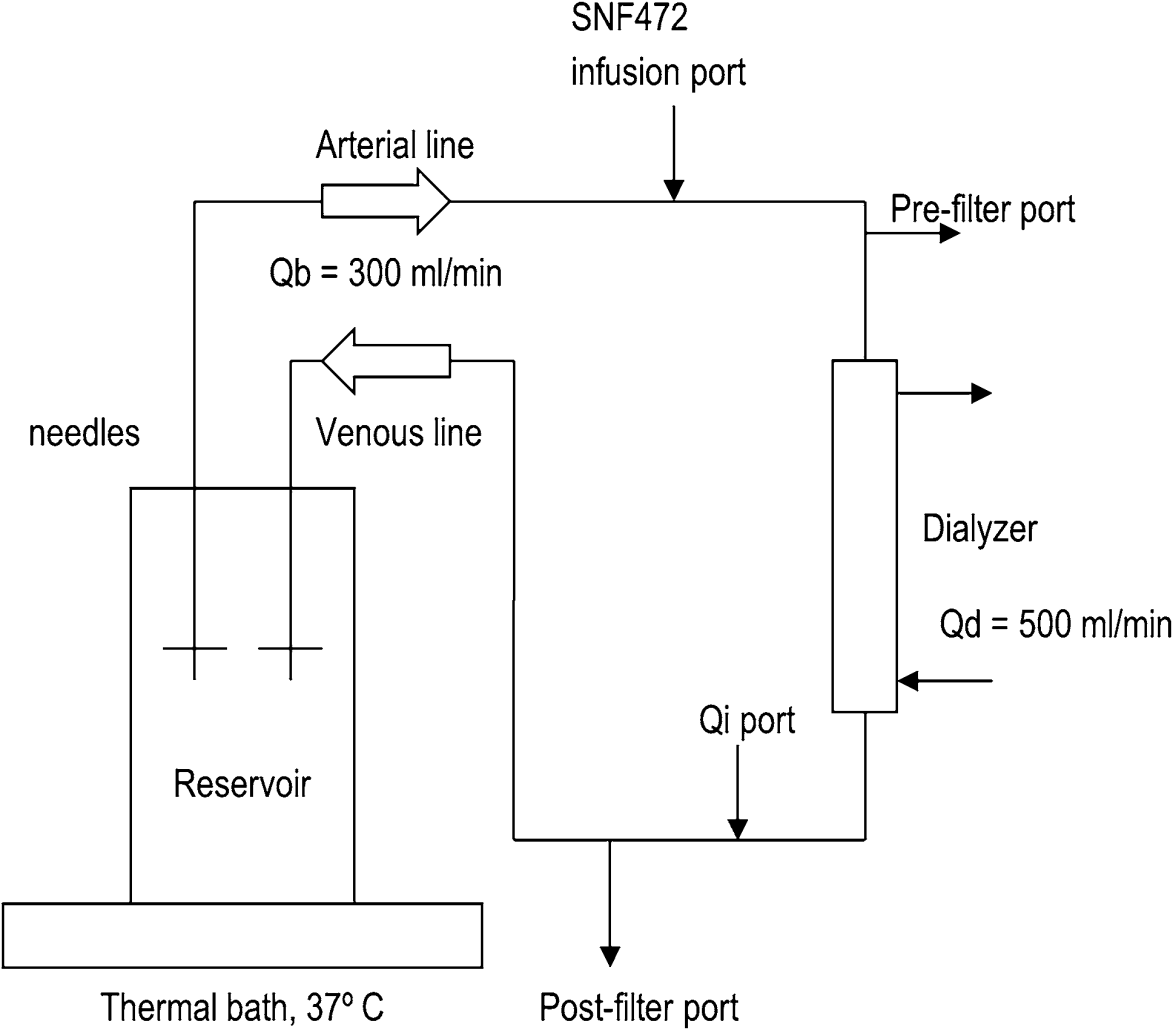
Scheme of the infusion system

*In vitro* experiments were performed using FMC 4008 (Hospital Son Llàtzer) and FMC 5008 (Hospital Clínic) dialysis devices. One litre of continuously stirred saline or heparinized whole blood (heparin sodium, 1000 U/l) kept at 37 °C was introduced in a reservoir forming a close-loop circuit (Fig. 1). AV-Set ONLINE-Plus BVM 5008-R blood lines (FMC) and 15G needles (BBraun) were used in 5008 monitors and AV-Set SRB-R blood lines (FMC) 2008/4008 in 4008 monitors. Creatinine was added to the reservoir to attain an initial concentration of 8 mg/dl. Known concentrations of SNF472 (10, 30 or 66.67 mg/l) were infused in the system for 20 min by the SNF472 infusion port by means of an infusion pump. Prior to SNF472 infusion, the system was left on HD or OL-HDF for 15 min in order to homogenize the initial levels of total and ionised calcium and check the performance by means of the creatinine clearance. In the 4-hour experiment, SNF472 was administered by bolus to a saline solution and the hemodialysis system ran for a total of 4 h. In the bypass experiments (except for the experiment with 66.67 mg/l SNF472 in blood), the device was also running in HD for 15 min prior to the beginning of SNF472 infusion in order to stabilize the system and increase the calcium levels at the saline reservoir, and then was switched to bypass mode just before starting SNF472 infusion.

Blood flow, Qb, of 300 ml/min, dialysate flow, Qd, of 500 ml/min and ultrafiltrate, UF, of 96 ml/h were used in both HD and OL-HDF experiments. In addition, a substitution flow, Qi, of 80 ml/min was used in the OL-HDF experiments. In HD experiments, Qi was set to 0 whereas in bypass mode, neither Qd nor Qi was used. Calcium concentration in the ACF3A4 dialysis bath was 1.5 mM.

Blood samples were collected in K_3_EDTA tubes from the pre- and post-filter ports at times 0, 5, 10, 15, 20, 30, 40, 50, 60 min from the beginning of the SNF472 infusion, except where stated otherwise. Samples were centrifuged at 3500 rpm for 10′ and the collected serum aliquots were stored at -80 °C until the SNF472 concentration was assayed. Additional blood samples were collected from the pre-filter port in serum collection tubes at times − 15, -7, 0, 7, 12, 17, 22, 60 min from the beginning of the SNF472 infusion for biochemistry determinations.

### In vitro stability of SNF472 in human whole blood

The *in vitro* metabolic stability of SNF472 in human whole blood samples was studied for 1 h at two SNF472 concentrations (2.5 and 15 mg/l) and up to 4 h at 15 mg/l SNF472. The samples were incubated at 37 °C under mild agitation and aliquots were extracted at appropriate time points. SNF472 was determined in the plasma fraction obtained after centrifugation from each incubated blood sample.

### SNF472 quantification by UPLC®-MS

SNF472 was quantified using the method described by Tur et al. [26]. The bioanalytical procedure involved purification and extraction by protein precipitation with TCA in the presence of EDTA. The supernatant was diluted with TEAA 50 mM and injected into UPLC®-MS system. Quantitative analysis was performed by tandem mass spectrometry in the selected ion monitoring. The molecular ion of m/z 659 ([M.]-) was followed for quantitative purpose and was obtained after negative electrospray ionization. The compound was analysed by gradient reversed-phase chromatography using TEAA 50 mM pH 9 and ACN as mobile phase.

### Creatinine, total and ionized calcium determination (HC)

Creatinine and total calcium levels were measured by molecular absorption spectrometry. Creatinine was determined using the ADVIA 2400 analyser while total calcium was measured by CPC method using the ADVIA 1800 (Chemistry System of Siemens Healthcare Diagnostics, Chicago, IL, USA). Ionized calcium determination was performed using EPOC*®* blood analysis card [28].

### Clearance calculations

Creatinine clearance, K_cre_ (ml/min), was calculated by means of the solution of a single compartment model [29]. Assuming slight variations in volume, the expected exponential decay of the concentration can be written as:

1 $$C\left( t \right) \approx C\left( 0 \right){\exp ^{ - \frac{{{K_{cre}}t}}{V}}}$$

where, C(0) (mg/dl), is the initial concentration and, V (ml), the system volume. The values of creatinine concentration were used to fit the K_cre_ parameter by means of the least-squares method from the Gnuplot software fitting function, implementing the Levenberg–Marquardt algorithm.

Clearance of SNF472, K_SNF472_ (ml/min), was calculated by two different strategies. The first one involved the same exponential fitting method for creatinine, using SNF472 pre-filter levels. The second one was performed by means of calculating Eq. (2) [30] at each sampled time:

2 $${K_{SNF472}}=\left( {100 - Ht} \right)\;{Q_b}\;\left( {\frac{{{C_i} - {C_o}}}{{{C_i}}}} \right)+UF\;\left( {\frac{{{C_o}}}{{{C_i}}}} \right)$$

where *Ht* is the % of haematocrit, and *C*_*i*_ and *C*_*o*_ are the concentrations at the pre-and post-filter ports, respectively.

As we measured *C*_*o*_ > *C*_*i*_ in several time intervals for SNF472, Eq. 2 was not useful to obtain realistic clearance values. The presented results are obtained from the exponential fitting.

## Results

**Fig. 2:**
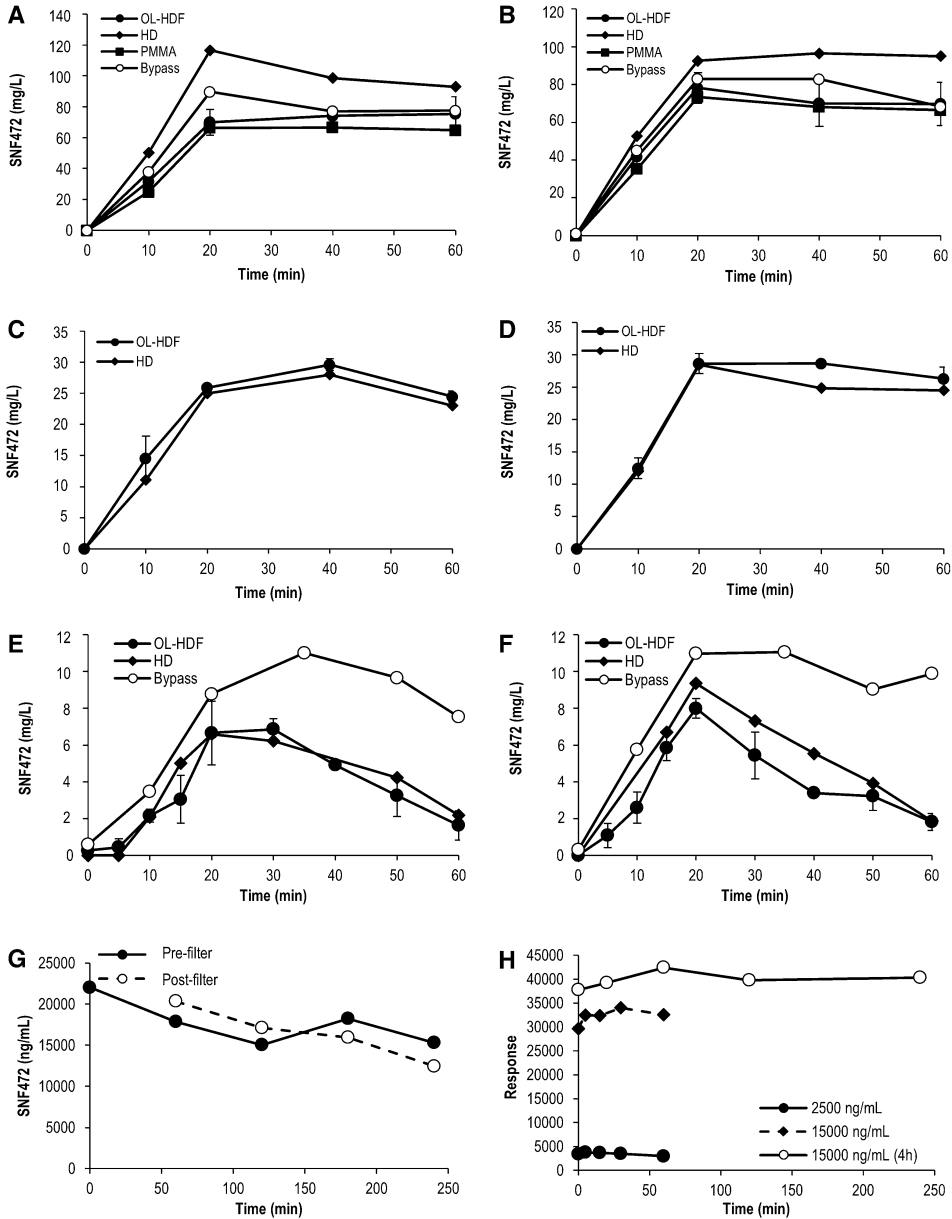
In vitro dialysability and stability of SNF472 under different experimental conditions. (A) 66.67 mg/l SNF472, pre-filter sampling; (B) 66.67 mg/l SNF472, post-filter sampling; (C) 30 mg/l SNF472, pre-filter sampling; (D) 30 mg/l SNF472, post-filter sampling; (E) 10 mg/l SNF472, pre-filter sampling; (F) 10 mg/l SNF472, post-filter sampling; (G) 30 mg/l SNF472 in saline with OL-HDF; (H) *in vitro* stability of 2.5 and 15 mg/l SNF472 in human blood at 37 °C. (A-F) SNF472 was infused at different concentrations for 20 min in 1 l blood. OL-HDF experiments performed in triplicate, mean ± SEM is represented HD and bypass experiments performed in single experiments. OL-HDF: online-hemodiafiltration; HD: hemodialysis; PMMA: polymethylmethacrylate membrane

SNF472 increased in blood while infused, reached a plateau and remained nearly constant when added at concentrations of 30 and 66.67 mg/l (Fig. 2A-D). The use of a polymethylmethacrylate (PMMA) membrane instead of a polysulfone (PS) membrane did not affect the dialysability of SNF472. There was no apparent loss of SNF472 when the system ran in bypass mode. However, when SNF472 was added at 10 mg/l its levels in blood increased up to 8 mg/l during the infusion but then dropped with estimated values of K_SNF472_ of 36 ± 3 and 17 ± 4 ml/min for OL-HDF and HD, respectively (Fig. 2E-F; Table 2). A longer experiment was performed with SNF472 at a final concentration of 30 mg/l subjected to dialysis for a total of 4 h (Fig. 2G). The results of the experiment confirmed that SNF472 dialyzed also at 30 mg/l but with a clearance so low that it was not detected in the 1-hour experiments when the compound was at or above 30 mg/l. An *in vitro* stability test was performed in human whole blood in order to discard SNF472 degradation during the hour of incubation. As observed in Fig. 2H, SNF472 is stable for up to 4 h in blood at 37 °C, at concentrations between 2.5 and 15 mg/l.

**Table 2:** Clearance values for creatinine and SNF472 infused in blood and saline

Experiment	SNF472 clearance (ml/min)	Creatinine clearance (ml/min)
OL-HDF – Blood^1^	36 ± 3	204 ± 23
HD – Blood^1^	17 ± 4	161 ± 17
OL-HDF – Saline^3^	7 ± 2	321 ± 35
HD – no calcium – Blood^2^	115 ± 6	171 ± 25
HD – no calcium – Saline^3^	80 ± 13	238 ± 7

Clearance of SNF472 was calculated by the exponential fitting method for creatinine, using SNF472 pre-filter levels. Results represent fitted parameter mean values ± asymptotic estimated error

^1^10 mg/l SNF472

^2^66.67 mg/l SNF472

^3^30 mg/l SNF472

**Fig. 3:**
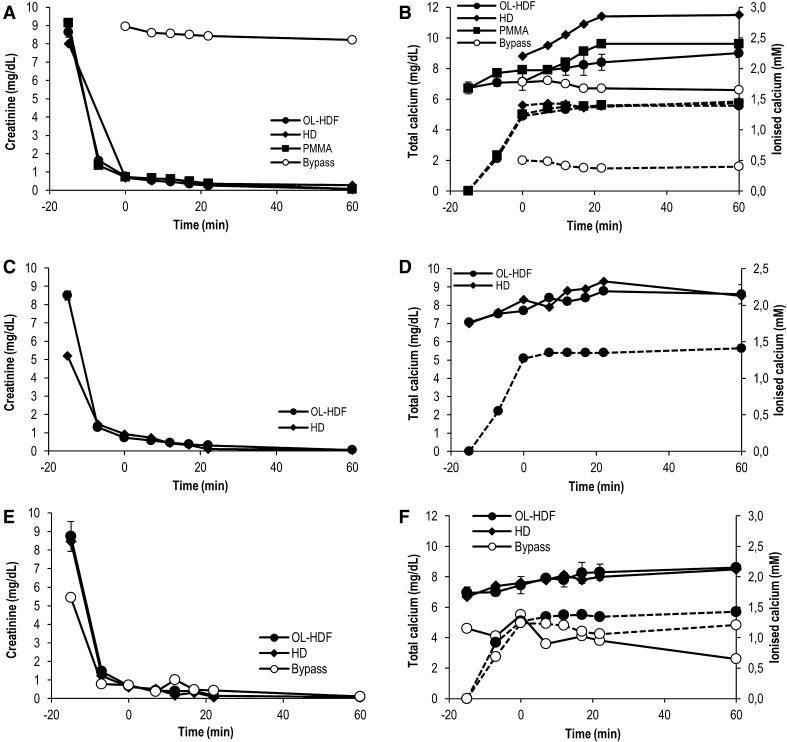
Creatinine clearance and SNF472 effect on calcium levels in in vitro dialysis systems. SNF472 was infused at different concentrations for 20 min in 1 l blood. (A, B) 66.67 mg/l SNF472; (C, D) 30 mg/l SNF472; (E, F) 10 mg/l SNF472. In (B, D and F) solid lines represent total calcium while dashed lines represent ionized calcium. OL-HDF experiments performed in triplicate, mean ± SEM is represented. HD and bypass experiments performed in single experiments. OL-HDF: online-hemodiafiltration; HD: hemodialysis; PMMA: polymethylmethacrylate membrane

Creatinine dialyzed with K_cre_ values of 204 ± 23 and 161 ± 17 ml/min for OL-HDF and HD, respectively (Fig. 3). Ionized calcium levels were low in bypass mode (25% of total calcium) but this was resolved when switching the system to dialysis mode, arriving to ionized calcium levels around 60–70% of total calcium.

**Fig. 4:**
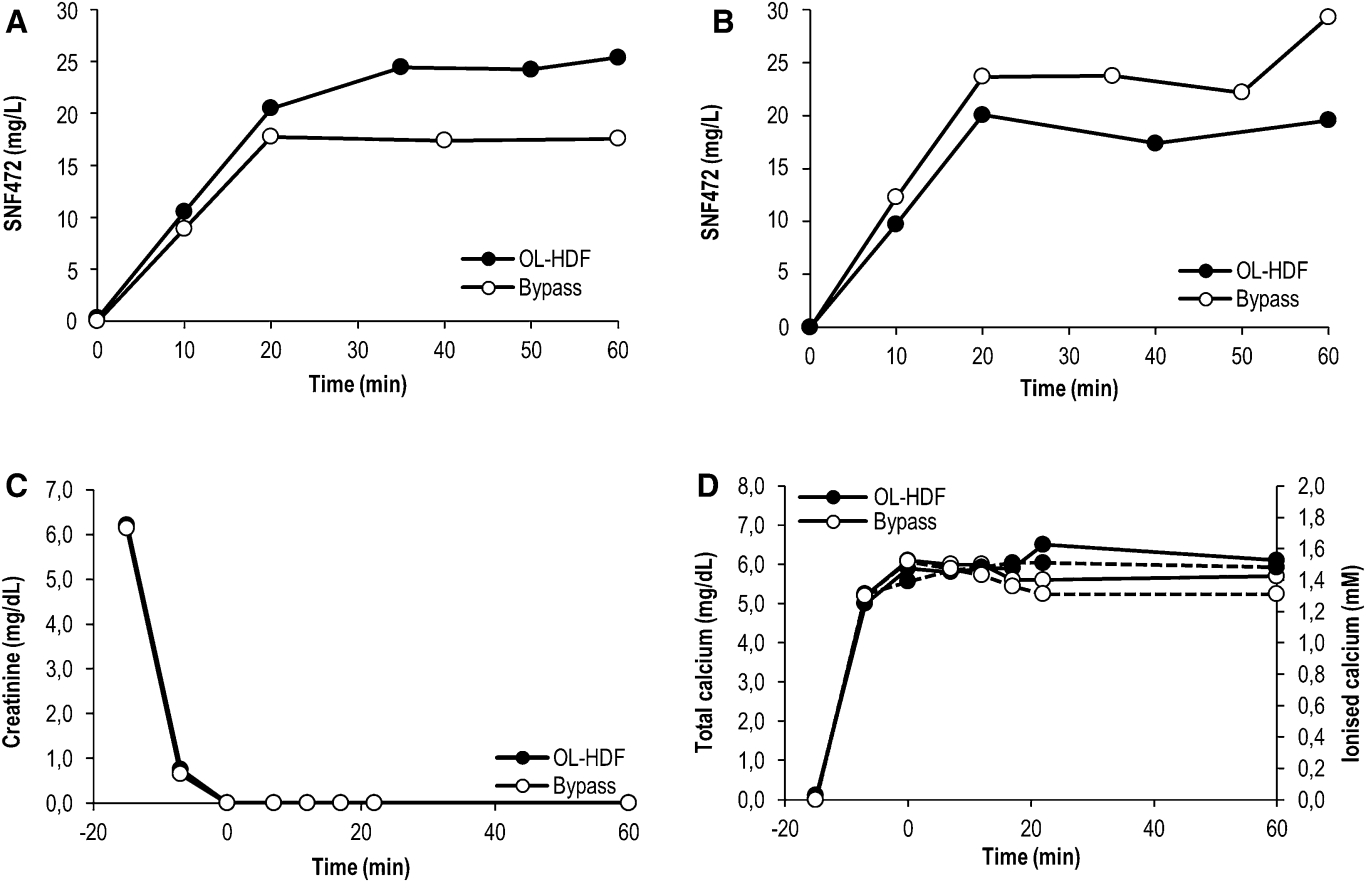
In vitro dialysability of 30 mg/l SNF472 under different experimental conditions in saline samples. SNF472 was infused at 30 mg/l for 20 min in 1 l saline. (A) Pre-filter sampling; (B) Post-filter sampling; (C) Creatinine clearance; (D) Total (solid lines) and ionized (dashed lines) calcium levels. OL-HDF: online-hemodiafiltration

In order to check if the lack of dialysability observed at high concentrations was due to interactions with blood proteins, the assays were performed in saline with 30 mg/l SNF472. As seen in Fig. 4A, B, SNF472 reached maximum plateau levels after infusion and these were maintained up until the end of the 60-min period. Creatinine correctly dialyzed in these conditions (Fig. 4C). In the bypass experiments (except for the experiment with 66.67 mg/l SNF472 in blood), the device was running in HD for 15 min prior to the beginning of SNF472 infusion in order to stabilize the system and increase the calcium levels at the saline reservoir, and then was switched to bypass mode just before starting SNF472 infusion. In these conditions a very slight chelation of calcium by SNF472 was observed (Fig. 4D).

**Fig. 5:**
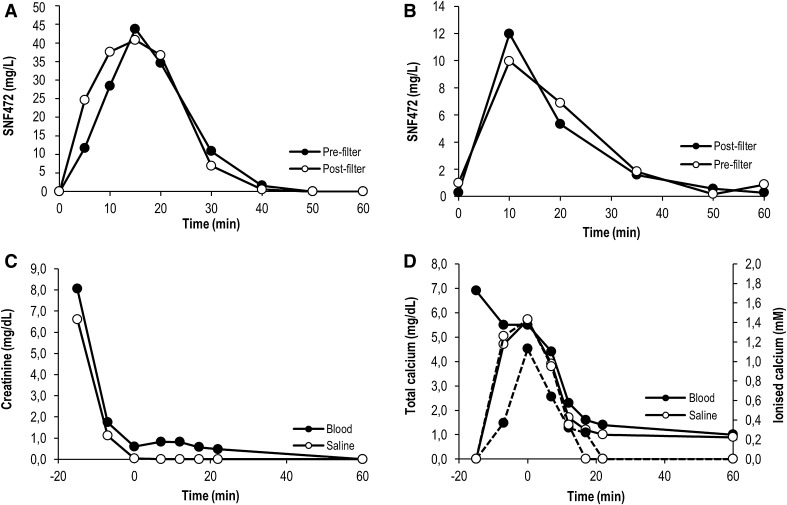
In vitro SNF472 dialysability in blood and saline in the absence of calcium in the dialysis bath. SNF472 was infused for 20 min at (A) 66.67 mg/l in 1 l blood or (B) 30 mg/l in 1 l saline; (C) Creatinine clearance; (D) Total (solid lines) and ionized (dashed lines) calcium levels

Finally, the experiments were performed using a calcium-free dialysate in order to study the possible effect of SNF472-calcium aggregates formation. The system was previously stabilized for 15 min in HD, and then this was changed to a calcium-free concentrate. SNF472 levels rose in blood for the 20 min of infusion while total and ionized calcium levels dropped (Fig. 5A, D). In the absence of calcium, SNF472 dialyzed in HD with a K_SFN472_ of 115 ± 6 ml/min for the 66.67 mg/l tested concentration and was undetectable after 50 min of study. A similar behaviour was observed when the assay was performed in saline in HD with 30 mg/l with a K_SFN472_ of 80 ± 13 ml/min (Fig. 5B, D).

## Discussion

Patients with CKD exhibit more CVC than age-matched healthy individuals, and patients receiving HD therapy are reported to have calcification scores over fivefold higher than matched individuals with established coronary artery disease but normal kidney function [31]. The presence of CVC is also a strong predictor of future cardiovascular (CV) events, CV death and all-cause mortality [32, 33], but there are currently no approved therapies indicated for the treatment of CVC. A number of potential therapies target the calcification process, but these are currently experimental and there are few controlled studies on which to base therapeutic decisions. There is certain evidence that treatments designed to control parathyroid hormone (PTH), Ca and P levels in patients with advanced CKD such as calcimimetics and phosphate binders may attenuate calcification progression in HD patients with secondary hyperparathyroidism [34–38].

The mechanisms by which soft tissue calcification in HD patients is triggered are not fully understood but disturbances in Ca and P metabolism and the down-regulation of endogenous inhibitors of calcification at early stages of the disease and its loss during dialysis seem to be involved [14, 15], together with the transdifferentiation of vascular smooth muscle cells (VSMC) towards an osteoblast-like phenotype [39, 40]. The development and acceleration of CVC is especially enhanced in patients undergoing dialysis, in which pyrophosphate seems to be lost through the dialysis system [10, 41]. Phytate is a naturally-occurring substance found in cereals but also present in mammalian cells and tissues at concentrations in the µM range [16] and in human plasma at concentrations below 0.5 mg/l [26]. Phytate is a potent endogenous inhibitor of hydroxyapatite (HAP) crystallization that acts by interfering the deposition of new calcium and phosphorus ions on the growing HAP crystals [42]. Animal studies with rat models of calcification have shown a promising effect of phytate on the inhibition of CVC *in vivo* [23–25]. However, the low MW of phytate and its high water solubility make it potentially dialyzable and the loss of this inhibitor during HD could additionally exacerbate the development of CVC. A recent publication hypothesized that the dietary intake of phytate could attenuate the development of age-related CVC [11], even at the early stages of CKD [43], and the accelerated CVC seen in HD patients could be partially explained by the loss of phytate and be counteracted by supra-physiological phytate plasma concentrations that cannot be achieved by the oral route due to the limited gastro-intestinal absorption. SNF472 is a novel experimental drug with phytate as its active principle which is currently in development for the treatment of calciphylaxis and the attenuation of CVC progression in HD patients. The intended posology of SNF472 is intravenous infusion during dialysis, in order to obtain supra-physiological phytate plasma concentrations which would produce its therapeutic activity inhibiting CVC.

In the current study, in an *in vitro* setting of HD we demonstrated that phytate dialyzes from blood with a low clearance and this dialyzation becomes especially relevant when the compound is present at low concentrations (around or below 10 mg/l). SNF472 was infused to the blood for 20 min until reaching the desired final concentrations (10, 30 and 66.67 mg/l) and then dialysability of the compound was followed for 40 additional min. Once the desired levels were attained, no significant loss was observed when the compound was added at 30 and 66.67 mg/l but significant dialysability was observed at 10 mg/l with K_SNF472_ values of 36 ± 3 and 17 ± 4 ml/min for OL-HDF and HD, respectively. This higher clearance observed in the OL-HDF system is comprehensible as this system applies a high convention at the filter, extracting the plasma water at a rate of around 80 ml/min which could enhance the depuration of the studied solute. The difference in dialysability found in the different SNF472 concentrations tested is explained by the low clearance of the compound, as the extension of the dialysis session to 4 h confirmed this low clearance also when SNF472 was present at 30 mg/l. In order to discard an effect of SNF472 degradation during the time of dialysis, an *in vitro* stability assay was performed and the results obtained confirmed that the observed loss of SNF472 was in fact due to dialyzation rather than to degradation. Although relevant dialysability of SNF472 was observed at 10 mg/l, it must be noted that during the infusion period the final desired levels were nearly attained and the clearance of the compound is almost 10 times lower than that of creatinine. This behaviour is similar to previous observations in other polyphosphate compounds such as pyrophosphate and bisphosphonates. The quantification of pyrophosphate levels in blood of HD patients both before and after HD treatment revealed a significant 32% decrease [10] in this substance content. Similarly, the measurement of pamidronate levels after a 2-h dialysis session revealed a 32% loss of this substance through the dialysis membrane with a clearance of 69.3 ml/min [27] whereas for clodronate [44] the reported values of clearance were 87.8 ± 16.2 ml/min in HD patients, both of them lower than that found in urea or creatinine but higher than the value we report for SNF472 (17 ml/min in HD). These results evidence that phytate is lost during dialysis with a low clearance, but considering the very low endogenous levels of phytate (around 0.5 mg/l) this low clearance can be enough to deplete circulating phytate in HD patients, thus exposing these patients to accelerated development of CVC. SNF472 is a novel approach to obtain sustained high phytate plasma levels in these patients.

Phytate has already been shown to bind to proteins [16], especially on the cell membrane of erythrocytes, without entering into the intracellular space due to the presence of high negative charge [45, 46]. Therefore, SNF472 in blood could be found bound to cell membrane or plasma soluble proteins and this would make its dialyzation difficult. The nature of the circulating proteins which bind phytate is not known, but the high negative charge of the compound points to unspecific charge–charge interactions, possibly through calcium ions forming phytate-calcium-protein complexes. However, the same results were obtained when experiments were performed in blood and in saline, so the possibility of protein binding being the main or only cause for the low dialysability of the drug was discarded and other mechanisms to retain SNF472 are likely involved.

The presence of six negative charges on the surface of the molecule also provides SNF472 with the capability to chelate divalent cations such as calcium. The formation of colloidal SNF472-calcium complexes with a characteristic size and charge could avoid their passage through the dialysis membrane pores. When experiments were performed without calcium in the dialysate, dialysability of SNF472 was evidenced after finishing the infusion, even at the 66.67 mg/l concentration, with K_SNF472_ of 115 ± 6 and 80 ± 13 ml/min in blood and saline, respectively. These results taken altogether point to the formation of SNF472-calcium complexes that would be multi-charged (both positively and negatively) and with a size enough to render difficult their dialyzation. Although the formation of these complexes implies a slight chelation of calcium by SNF472, no significant decrease in ionized calcium levels was observed, which should be attributed to the compensating effect of the dialysis bath in the calcium concentration. The small clearance of SNF472 as well as the slight calcium chelation could be explained by assuming the formation and disassociation of the calcium aggregates in a dynamical regime, which leads to a low dialysability of the compound due to its size and charge. However, in the calcium-free experiments, levels of SNF472 increase to a lower than expected level, and the maximum peak is reached before the total infusion of the compound. The amount of calcium in the first minutes of this experiment leads to a dynamical formation and disassociation of calcium aggregates while there is enough calcium at the reservoir. When the calcium depletion of the reservoir reaches a threshold level, aggregates cannot be reassembled, leading to free SNF472 to be dialyzed, even while the compound is still infused in the system. However, as a rich-calcium dialysate was formerly used both in HD and OL-HDF treatments, slight chelation of calcium levels of the dialysis bath does not represent a significant cation depletion. Therefore, these results evidence that in addition to protein binding, SNF472 binds to calcium to form colloidal aggregates that cannot cross the dialysis membrane.

In conclusion, SNF472 dialyses out with a low clearance, evidencing that the endogenous crystallization inhibitor phytate is lost during dialysis. The low dialysability compared to other structurally similar molecules seems mainly due to the formation of complexes with calcium. The intravenous administration of SNF472 during the dialysis session could provide the means to increase phytate levels to supra-physiological values with the potential to slow down the progression of CVC in ESRD patients. When SNF472 is administered through infusion, its levels increase in blood during the infusion period and dialysis does not prevent from attaining potentially therapeutic levels, as the levels reached at concentrations of 10 mg/l are the ones that showed efficacy in the *in vitro* and *in vivo* efficacy tests [47] as well as in pharmacodynamic measurements performed in Phase 1 clinical trials with SNF472 [48]. The chelating effects of SNF472 on calcium are compensated by the calcium in the dialysis bath, so hypocalcaemia in the dialysis setting is unlikely.
